# Promoting Participation in Daily Activities Through Reablement: A Qualitative Study

**DOI:** 10.1155/2020/6506025

**Published:** 2020-01-27

**Authors:** Trine A. Magne, Kjersti Vik

**Affiliations:** Department of Neuromedicine and Movement Science, Norwegian University of Science and Technology Trondheim (NTNU), 7036 Trondheim, Norway

## Abstract

A well-known prediction is that the growing elderly population will place a strain on our healthcare systems. At the same time, healthcare is becoming increasingly patient-centered and individualized, with the patient becoming an active participant rather than a mere object of healthcare. The need for change may be met by using a reablement service, utilizing the rehabilitation mindset through home-based services. Rehabilitation and reablement aim to provide opportunities for individuals to participate to a maximum of their potential. This study is part of a larger research project exploring different aspects of reablement in municipalities. It aims to describe how older adults engage in daily activities within the context of reablement and to explore participation in daily activities. A qualitative design was chosen, and the study is explorative in nature due to limited research on participants' experience with reablement. Ten older adults age 70 to 94 years old were recruited and interviewed. The interviews were transcribed verbatim and analyzed using systematic text condensation (STC) strategies. This study provides insights on how older adults experience participation in daily activities and important aspects for performing these activities and living independently as long as possible. Based on the older adults' experiences, three main themes were identified when receiving reablement. First, what to achieve with reablement and feeling a sense of security to participate in daily activities. Second, how to carry out wanted activities using different skills and last, how the social network is important for enabling active living. This calls for healthcare workers to address and facilitate these in reablement. Our findings show the importance of collaborating with the social network and strengthening participation in daily activities to establish and develop existing reablement services.

## 1. Introduction

A well-known prediction is that the growing elderly population will place a strain on our healthcare systems [1, 2]. At the same time, healthcare is becoming increasingly patient-centered and individualized, with the patient becoming an active participant rather than a mere object of healthcare [3]. Instead of relying on help to perform daily activities, e.g., to shower or prepare meals, the service user is becoming the driving force in receiving health care to fit their needs and manage on their own [4]. In addition, the service providing care needs has shifted from a one-sided focus on disease and functional limitations to a more balanced approach seeking to enhance the elderly person's resources, skills, and participation in daily activities. In Norway, this has called for service innovation in municipalities [1, 5, 6].

The need for innovation can be met by implementing reablement service, by utilizing the rehabilitation mindset through homecare services. Rehabilitation aims to provide equal opportunities for people to participate and take part in their daily lives to the best of their potential. Thus there are limited descriptions of the theoretical background of reablement, reablement seeks to improve health by participation in everyday activities at home and in the local community, and to reduce the need for ongoing service support by focusing on personal goals and needs. Even though there are many definitions [6] no specific definitions of reablement are agreed upon [7]. In this study, “reablement” is understood as “an early and time-limited home-based intervention with emphasis on intensive, goal-oriented, and interdisciplinary rehabilitation for older adults in need of rehabilitation or at risk of functional decline” ([8], p. 1582). In addition, it is understood as “an intervention process that aims to retain, regain or gain skill to promote desired independent living” ([9], p. 574), consequently reablement also will enhance participation in daily activities.

Even though there are key concepts to reablement, such as time-limited service for older adults with reduced functioning, municipalities also establish their own service model based on the population demands and resources within the specific municipality. Reablement is often provided by homecare services or community-based teams consisting of nurses, occupational therapists, physiotherapists, and home trainers such as auxiliary nurses and assistants trained in skills required to assist in rehabilitation [10].

The increase in reablement services has called for research on the impact of change this service has given for older adults, service providers, and next-of-kin, as well as the organization of services. For example, some studies have reported on the implementation of reablement [11–13], others on the experience of service providers [1, 11] and next of kin [14]. These research results and experiences about reablement services in municipalities are provided most from the professionals' perspective, and there is still limited knowledge about older adults' experience with implementation during the period they receive reablement services.

In studies that have been published about older adults' experiences, Hjelle et al. [8] found that the driving forces for reablement were both intrinsic and extrinsic motivational factors. Intrinsic factors represented as willpower and responsibility, and local environment in addition to reablement team as important extrinsic factors. This is in line with Jokstad, Landmark, Hauge and Skovdahl [15], who found that believing in one's own possibilities and resources was important for reablement. Furthermore, Tappes-Lomax and Hawtorn [16] focus on the complexity of rehabilitative need and the influence of the setting. mann's [13] study indicate that the setting, adapting to environments are of importance for optimizing capacity for older adults undergoing reablement and giving them a push for physical strengthening and building confidence. These studies focus on individual strategies.

Since reablement aims at participation in daily activities, there is still a need to expand knowledge of how older adults experience their opportunities to participate in daily activities within the context of reablement services. This study is a part of a research project collaborating with several municipalities in Norway to investigate different aspects of reablement service from different stakeholders.

## 2. Methods

### 2.1. Aim of the Study

The specific aim of the study is to describe how older adults engage in daily activities within the context of receiving reablement and to explore participation in daily activities. Therefore, a qualitative design was chosen. The study is explorative in nature due to limited research on participants' experience within reablement.

### 2.2. Settings

In this study, in an urban and rural municipality, reablement services was carried out with two different service models for organization and implementation. In the urban municipality, reablement is organized as part of the homecare services using homecare workers supporting the reablement interventions, while the therapists (e.g., occupational and physical therapists) supervised homecare workers. In the rural municipalities, the therapists and homecare workers worked together in teams within homecare services. Even though there are different service models, the main focus of the service provided remains the same and therefore this difference is not explored further. The main focus in both municipalities is assisting and supporting older adults to participate in everyday life through reablement. The older adults are in charge of setting goals focusing on their wish for participation in daily activities and how to regain and gain skills to achieve these goals. The target users of the service were also the same in both municipalities, adults over the age of 65, already receiving homecare.

### 2.3. Participants and Data Gathering

Older adults that had been included to receive reablement service were potential study participants. The municipalities had criteria to receive this service. These criteria were similar in both municipalities and included for example the ability to collaborate with the healthcare service providers and take part in the reablement process. Healthcare workers were informed about the research study and were in charge of the recruitment process. Convenient sampling strategies were used [17], as the recruiter payed attention to diversity among the participants to seek variation in age, gender, living accommodation, and marital status. As baseline for inclusion, the older adult had to be included in the reablement service (within the municipalities' inclusion criteria) and being able to communicate in an interview estimated to last for 30 to 90 minutes. Participants with memory deficits or impaired language that affected their communication skills were not included. These issues were based on the recruiter's assessments. However, the healthcare workers paid attention to this when recruiting.

Ten older adults between 70 and 94 years old were included (see Table 1), and the participants' characteristics are presented. All recruited participants took part in this study, nobody withdrew. The interviews were carried out while they received reablement service, varying between the first and fifth-week from start-up.

The first author conducted the interviews in the participants' home and recorded the interviews with informed consent. Data collection was carried out with one participant at a time. A semistructured interview guide was used and the questions were developed based on the aim of the study focusing on the participant's experience of participation in daily activities, e.g., “What have been/are your main activities in your life and how did/do you participate in these?” and “How do you experience participation in daily activities while receiving reablement?”.

Collaboration with the participating municipalities did not include any economic benefits on either part. This study was approved by the Regional Committees for Medical and Health Research Ethics in Norway (REK midt 2014/2028). Participation was voluntary, and the participants was informed that they could withdraw from the study at any time.

### 2.4. Analysis

A scientific assistant transcribed the interviews verbatim. Transcribed data were then analyzed by the author using systematic text condensation (STC) strategies presented [17]. These strategies are based on Giorgi's psychological phenomenological analysis and describe the qualitative analysis process using four steps, starting with acquiring a total impression of the transcribed interviews and identifying preliminary themes.

Because the participants lived in different municipalities, the interviews were first analyzed as separate groups, and the text was structured into coded meaning units, e.g., family and being independent. Later it became evident that the categories identified were similar and therefore further analyzed as one group. In the third step was sorting decontextualized units in the coded themes for each participant, e.g., performing activities and motivation. The participants were given coded names to ensure they later could be identified in the original group, enabling the possibility of discussing the impact of differences in living in municipalities.

Finally, in the fourth step the analysis was finalized by synthesizing the condensed text into descriptions and concepts [17], e.g., exploring skills and sense of security.

During the analysis, three themes “Achievements within reablement context and feeling sense of security,” “Performing wanted daily activities using different skills,” “Social network, a key to participation,” were identified with regards to the study aim.

## 3. Findings

Based on the empirical data, the three main themes represent the participants experience within the context of reablement and are presented in the results. Theme one, “Achievements within reablement context and feeling a sense of security,” identifies important factors for the participants when receiving reablement. The second theme “Performing wanted daily activities using different skills,” summarizes daily activities and how to carry them out. The last theme, “Social network, a key to participation,” describes how friends and family participate in the participants' lives and how the home environment and neighborhood enable active living. This is an important factor when undergoing reablement.

### 3.1. Achievements Within a Reablement Context and Feeling a Sense of Security

An interesting finding was that the older adults changed their opinions on what to achieve related to daily activities during the reablement period. The response varied on what they believed they could achieve regarding their daily activities through the interventions. Understanding the purpose of reablement seemed to become clearer further into the process. Newly started reablement participant Karen responded, “*No…I don't know if I become better. That I have little faith in.*” Beate, also having received reablement for only one week, said, “*I can't comment on that yet, because it is so new. However, I believe it helps*”. Toward the end of reablement, Ole explained a clearer understanding of possible achievements with reablement. “*But of course, I become better when I've been doing activities*”. The variations in statements on the importance of reablement did not indicate whether the participants' experiences corresponded with the intentions of the service given by the municipality. Even though there was no clear understanding of the intervention, the participants' reflections on the reasons they received the service illustrated why they needed it to participate in daily activities. As Hans said, “*I started reablement probably because I was sitting too much*”. When Aksel was asked what he would do if he had not received this service, his response was, “*I had still been sitting here (in his recliner)*”.

In order to engage in daily activities again, the importance of a sense of security in performing wanted daily activities, were described as important achievements for participating in daily activities and living independently. The participants gave example such as; walking to the grocery store, with not fear of falling any more, or to avoid sitting too much or becoming more confident in moving outside of their home. Feeling a sense of security motivates them to become active and seemed to be an important factor for carrying out daily activities.

Participants also highlighted the importance of becoming stronger to manage daily activities and physical function being another key factor. As Kristin said, *“I would not have been this physically fit if I had not had reablement. No, I must be honest about that. It does me well, really*”. Exercises instructed by the homecare workers were said to ensure participants of being able to perform wanted activities and motivated them to think of the need to become more active in their lives. However, exercises introduced to improve physical function through reablement were said to be too easy and not inspiring. As Beate said, *“If I regained the desire to work…if there was hope of that, then I would become very happy*.”

The participants said reablement was a positive experience. Even though the exercises were experienced as not inspiring, the participants agreed that having someone there, watching, when they performed physical exercises and daily activities restored their confidence to become active, and receiving feedback was of importance. Marie commented on this by saying, *“…last time, I was praised a little on my balance, which I'm not so good at. I don't know if it was only to comfort me, but at least I was praised*”.

A third factor, the participants told about learning new or smarter ways to perform daily activities. This was promoted by the homecare worker being there with them and providing feedback. Focusing on abilities to perform and participate in activities, the homecare workers' support influenced the older adults' motivation to perform the activities by themselves later. One participant explained how she now takes the bus downtown alone to meet with friends after taking the bus with a homecare worker to assure her she could manage, something she had not done in years. Others explained that participating in local arrangements involving their social network motivated them to work on different skills to be able to take part in the activity. The participants pointed out that trying reablement and achieving the confidence to perform daily activities was important because they did not want to bother their network.

### 3.2. Performing Daily Activities Using Different Skills

The participants described important activities they wanted to participate in such as walking, meeting friends, being with family and traveling independently to, for example, the grocery store or a cafe. Furthermore, important weekly activities were meeting friends at cafes and sharing a meal together. One participant mentioned being able to walk with friends or neighbors on a regular basis as important. Other activities of interest were traveling, solving crossword puzzles, watching television, and activities carried out at home. Different strategies were used to regain skills and thus become more confident in performing these activities.

Participants described different strategies such as using more time to perform an activity, dividing activities over several days or using assistive devices (e.g., walker), depending on the motivation to perform the desired activity. Dividing an activity over several days was explained by Kristin as, “*I do daily chores, not all in one day. No, I sort it *(cleaning the apartment)* somehow day by day. So, on Saturday I wash windows*.” However, if it became too difficult, they would avoid doing the activity. As Marie said, *“I can think 'tomorrow I'll do it' *(shopping for groceries).* However, when the morning comes, I won't do it. But I want to try, really I do*”. If different strategies did not work, and the activity was too hard to perform, family, friends or neighbors helped the participants. As a last resort, they sought help from the homecare service if they did not want to burden their network.

In general, the participants were satisfied with the amount of activities they wanted to perform, and some did not long to participate in new activities. As Ole described it, *“I do very little each day; however, I'm not bored*”. Even though they did not long for more activities, the ability and motivation to participate were the reason they saw the importance for focusing on daily activities in reablement. Karen exemplified this when she talked about getting dressed in the morning. “*This *(reduced physical function)* is something you just have to get used to. It is nothing to do about it, that's for sure…I keep on trying, until I get it done…I think it is good to manage it by myself*.”

### 3.3. Social Network, a Key to Participation

Even though reablement services focus on the individual's engagement and skills, the participants told about the importance of having their families close by for the opportunity to engage in daily life. They described the family as helpers and motivators, both for participating in social settings, and living an active life. Their children and grandchildren play a vital role in helping with daily activities such as shopping for groceries, paying bills, house maintenance, and driving to the doctor. To stay connected with their network, they would talk on the phone, use Skype, and have friends and family come to visit. However, they did not want to be a burden, and therefore they did not invite as much as they wanted or call as often as they wished to.

Interestingly, participants described the importance of their social network in connection to reablement more than the professionals. The participants' examples gave insight on differences in the roles in the families to promote engagement. For example, family living close to the participants in our study became helpers (e.g., household chores and transportation), whereas family members living far away were important for social contact (e.g., weekly talks on the phone or through Skype). As Beate said about her family, *“I manage pretty well by myself. It's only when I need to go to the doctor or do something special…I have my children, who live here, to help me*”. Neighbors living close were also important for looking after each other, walking together and meeting socially, as well as helpers when needed for, e.g., snow shoveling and other house maintenance chores. Marie described her neighbor with *“…her on the fourth floor, she visits me either at noon or in the afternoon several times a week. She sits here and talks…we take care of each other*”. Most participants had or have had homecare service for several years before receiving reablement. Nevertheless, none mentioned the staff as part of their social network. In need of specialized help to shower weekly or managing medicine, the homecare service provided expert help. This was only necessary when, for example, physical function changed, or sense of security was challenged and not seen as social contact.

The wish for living at home if possible and not moving to, e.g., a nursing home was important. The participants argued that living at home, everything was familiar to them; they have routines for e.g., doing laundry and making meals, and familiar things gave them the needed sense of security. They also argued that social network was close and the most important factor to enable active living and participating in daily activities.

## 4. Discussion

This study aimed to describe how older adults engage in daily activities within the context of receiving reablement and to explore participation in daily activities. One of our main findings suggest that within the context of reablement there is a potential to assess skills to participate and perform different activities in order to personalize the service and reach the older adults' goals. Another main finding suggest that there is a need for service providers in reablement to pay attention to the older adults' social network. The network having different roles when enabling the older adults to participate in different daily activities.

Even though there has been an increase in research on reablement [12], due to the variety of different understandings and organization of the service, more research is needed. In addition, as Rostgaard et al. [11] point out, there will be an increasing demand for greater choices, more personalized service, and better quality in homecare support. There is still limited knowledge of how older adults experience participation in daily activities within the context of reablement and our study can contribute to increase this gap in knowledge.

Cochran et al. [12] point out the importance of promoting a shared understanding of reablement within a service to ensure a shared understanding and expectations between the service user and provider. This is particularly important when implementing reablement service during an already well-established homecare service. Our findings suggest that the sense of security and social network are important for living independently as long as possible. By focusing on physical function (using different skills and strategies), and being supported by the homecare worker, older adults become confident in performing daily activities. This enables active living.

In reablement, the older adult collaborates with the healthcare worker to retrain, regain or gain strategies to manage everyday life as independently as possible [9]. Studying the older adults' experience after receiving reablement, Hjelle, Tuntland, Førland, and Alvsvåg [8] pointed out the need for follow-up programs to maintain being active, e.g., using strategies. In our study we found that it is important to seek knowledge on what strategies the older adults use and facilitate these within a reablement context. This knowledge contributes to the understanding of how older adults experience everyday activities and to support healthcare workers as to what to address the older adult who participate in these activities.

### 4.1. Enabling Participation in Everyday Activities

In agreement with the definition given by Aspinal et al. [9], where “retain, regain, and gaining skills” are important in the intervention process, our study adds knowledge on how this is experienced when wanting to participate in daily activities. The older adults exemplify how they use strategy to divide an activity over several days to manage it, and for example describing how one may use several days to clean her apartment.

Our study shows the importance of focusing on strategies and abilities in reablement. The participants highlight how to use different strategies and abilities in performing different activities and how healthcare workers are needed as supporters. One of the participants described how she was praised for her performance and how it comforted her. With reablement being a “time-limited” and “person-centered” service, Cochrane et al. [18] also highlight that facilitating performance of important daily activities as expressed by older adults is the main task in reablement.

However, it is important to pay attention to why some need help and what type of skills are needed to perform different activities. Petterson and Iwarson [19] describe a lack of attention to aspects regarding the meaningfulness of a specific activity. This suggests paying attention to activities of interest and, more specific, finding the motivation to perform wanted activities. Even though not investigated in this study, our findings add to the question of what should be focused on during reablement as older adults tend to have different motivations for performing different activities.

Tuntland et al. [6] state that having a goal-oriented approach is essential to succeeding with reablement, and the healthcare worker needs to facilitate setting goals that focus on daily activities. The older adults in our study varied both in reported activities to perform and the importance of goalsetting. However, they focused on the need to improve physical function as well as the need for a sense of security to be able to participate in wanted activities. Some of the older adults in our study were given, as part of the reablement service, the same exercise program to perform regularly. The participants in this study reported this program to be too easy and not inspiring, which suggests a need for individualized programs utilizing their abilities to motivate them to do the exercises. Furthermore, the healthcare workers' role in motivating and establishing a sense of security is important for older adults to perform the exercises and wanted daily activities independently. Participants said being with the healthcare worker was also important while performing activities that were essential for achieving the ability to participate in everyday activities. This may suggest that the focus on enabling performance of everyday activities should be both on promoting skills to support and facilitate the older adults when exercising and in performing activities. As Cochrane et al. [12] emphasize, in reablement it is important to work toward agreed goals that are designed to maximize independence and confidence.

Legg et al. [7] state that a lack of mutual understanding of the nature of rehabilitation among healthcare providers is of concern when working with a service user. This leads to the question of how this affects the collaboration with the person receiving the service. Even though this question was not addressed in our study, there is a concern that it influences implementation. When the older adults were asked about understanding the reablement service, our results suggest there is a change in understanding the intention for undergoing reablement. The participants become more confident of the intention toward the end of the service. In our study, all the participants already had homecare service before receiving reablement, and some received homecare during reablement; therefore, understanding the difference might be difficult. Because there is a difference between traditional homecare service and reablement service [12] evident in the municipalities participating in this study, it might cause the older adults in our study to use more time to understand the difference.

### 4.2. Strengthening Social Network

Turcotte et al. [20] highlight that participants need to involve daily activities as well as social activities. In our study, it became evident that the older adults' social networks played an important part in performing daily activities. Furthermore, the social network plays a different role in supporting the older adults' feeling a sense of security and being able to live an independent life. This is in line with findings among older adults who receive services in a municipality [14, 21, 22].

Findings from our study suggest that social networks may play different roles. Family and neighbors/friends are an important part of the older adults' life and contribute by helping the older adult in managing daily activities as well as contributing to social interaction. Living close to the older adult, the social network becomes helpers and living further away, more as social contact. This could be interesting to investigate further.

Furthermore, the different experiences with the social network indicate the importance of addressing this in reablement. Focus on strengthening the cooperation between the older adult and the social network seems to be a key element in reablement. This indicates that the homecare worker and/or therapist involved in reablement has an important role in facilitating such cooperation. Thus, involving the social network can be challenging due to, e.g., the older adults not wanting to become a burden or differences in meanings when it comes to the older adults' needs or goals. This was not investigated in the current study. The importance of addressing this matter leads to more research.

### 4.3. Methodological Considerations

One of the strengths in our study investigating participants who received reablement is that our findings agree with other studies related to exploring reablement. For example, our findings addressing the importance of enabling participation in everyday activities and strengthening the social network. Another strength is that the authors collaborated closely during the analysis process, and findings were discussed within our research group, this strengthen the trustworthiness of the study.

In-depth interviews were used to investigate the older adults' experience within the context of reablement service and cannot be generalized, however the findings in a qualitative study can point out certain aspects that also can be found in similar population of older adults, for example the importance of family, and to be encouraged by the professionals.

The inclusion criteria aimed for a variation in age, gender, and living conditions in order to have a variation of experience, and the participants were included to fulfill these criterions. Since the health care workers were responsible for the inclusion there is a danger that they “picked” out just participants who were positive to the services. But based on our findings the participants talked about positive and negative aspects of receiving reablement, we saw therefor no reason for this to influence data collection. Furthermore, due to the question of interest, exploring experience participating in daily activities, the participant had nothing to gain by participating in this study other than contribution on experience. The interviewer did not take part in the older adults' reablement process and had no prior engagement in the provided service.

Both authors are occupational therapist by profession, and our background have helped to form the aim of the study focusing on engaging and participating in daily life, as well as having a special interest in exploring the experience of activities. This may be a bias in the analysis. On the other hand, turning to research about older adults receiving services confirm how this engagement in ordinary daily activities are of importance in reablement [23].

Still a strength of the study is as the second author is an experienced researcher related to rehabilitation/reablement of older adults living in their homebased environment, the first authors' experience is mainly with rehabilitation in a hospital setting. Taken together these differences formed the analysis process by asking questions and not jump to conclusions without discussion.

### 4.4. Implications for Practice, Research, and Policy

The strength of the study is that it was initiated by the municipalities and therefore ensuring the relevance for practice.

There is an urgent need to address how home-based services can meet the challenges posed by the increased number of older adults in need of home care. Current services will not be able to meet the increasing pressure without evidence-based innovation in work methods and organization. Increased social network involvement and stimulating participation among older adults may be key elements in a strategy to improve quality and reduce passive services.

There is a need to critically examine current work methods, organization of services, as well as the role of the user and the social network, to lay the groundwork for a future healthcare service that is better able to promote independent living by managing daily activities if possible.

## 5. Conclusion

In this study, participants' understanding of reablement developed during the intervention. The older adults expressed the importance of feeling a sense of security when performing activities, and they gained knowledge of how being active to participate in daily activities supports being independent. Social networks play an important role in enabling active living. Taken together these conditions are of vital for both health care professionals and managers to be aware of when developing and implementation services for the coming generation of older adults and taking our findings into consideration this may improve and maintain health among older adults.

The interviews were conducted in the older adults' homes, allowing the opportunity to also observe the performance of activities at home. Even though these observational data were not included in this study, it could be interesting to investigate how activities are performed in a reablement context.

## Figures and Tables

**Table 1:** Participants' characteristics.

Participants	Gender^a^	Age	Week^b^	Living accommodations	Marital status
P1 (Hanna)	F	94	2	Farmhouse	Widow
P2 (Ellinor)	F	92	3	Farmhouse	Widow
P3 (Aksel)	M	90	4	Townhouse	Widower
P4 (Hans)	M	91	2	Sheltered accommodation	Widower
P5 (Karen)	F	87	1	Sheltered accommodation	Widow
P6 (Peter)	M	83	1	Apartment	Widower
P7 (Beate)	F	83	1	Apartment	Widow
P8 (Ole)	M	82	5	Apartment	Married
P9 (Marie)	F	87	4	Apartment	Single
P10 (Kristin)	F	70	4	Apartment	Widow

^a^Gender: F (female) and M (male). ^b^Week: week(-s) of receiving reablement before interviewed.

## Data Availability

The data used to support the findings of this study are available from the corresponding author upon reasonable request.
